# Immunohistochemical Expression of CaSR, VDR, and AMA in Parathyroid Tumors in Relation to Hypercalcemia Severity in Primary Hyperparathyroidism

**DOI:** 10.3390/cimb48080766

**Published:** 2026-07-28

**Authors:** Anna K. Eremkina, Dariya A. Pastuhova, Anastasiia P. Pershina-Miliutina, Anna M. Gorbacheva, Hanum V. Bagirova, Liliya S. Urusova, Natalya G. Mokrysheva

**Affiliations:** 1 Department of Parathyroid Pathology and Mineral Disorders, Endocrinology Research Centre, Dmitry Ulyanov St., 11, 117036 Moscow, Russia; a.lipatenkova@gmail.com (A.K.E.); gorbacheva.anna@endocrincentr.ru (A.M.G.); 2 Laboratory of Pathomorphology, Endocrinology Research Centre, Dmitry Ulyanov St., 11, 117036 Moscow, Russia; pastuhova.dariya@endocrincentr.ru (D.A.P.); liselivanova89@yandex.ru (L.S.U.); 3 Digital Transformation Department, Endocrinology Research Centre, Dmitry Ulyanov St., 11, 117036 Moscow, Russia; oa11111998@gmail.com; 4 Endocrinology Research Centre, Dmitry Ulyanov St., 11, 117036 Moscow, Russia; mokrisheva.natalia@endocrincentr.ru

**Keywords:** primary hyperparathyroidism, hypercalcemia, immunohistochemistry, parathyroid tumors, CaSR, VDR, cinacalcet

## Abstract

**Background**: Primary hyperparathyroidism (PHPT) is usually caused by parathyroid tumors, resulting in hypercalcemia due to excessive PTH secretion. While disease severity correlates with calcium blood levels, the molecular mechanisms driving clinical variability remain unclear. The role of the calcium-sensing receptor (CaSR), the vitamin D receptor (VDR) and parathyroid cells mitochondrial activity in the pathogenesis of hyperparathyroidism is of particular interest. **Methods**: This retrospective study included 96 patients with PHPT who underwent parathyroidectomy. Patients were stratified into four groups according to the baseline albumin-corrected serum calcium assessed at diagnosis, before the initiation of calcimimetic therapy (≤2.8; 2.8 < Ca corr. ≤ 3.0; 3.0 ≤ Ca corr. < 3.5; Ca corr. ≥ 3.5 mmol/L). Immunohistochemical expression (IHC) was estimated using CaSR, VDR, and antimitochondrial antibodies (AMA). Antibodies to VDR (GeneTex), CaSR (5C10, GeneTex), AMA (113-1, BioGenex) were used. Kidney tissue served as a positive control for CaSR and VDR antibodies, and liver tissue was used as a positive control for AMA. Statistical analysis was performed using the Statistica v. 13.3 software package (TIBCO Software Inc., Palo Alto, CA, USA; 2017). **Results**: Strong CaSR expression was observed in 95.8% of tumors, whereas VDR expression was reduced in 46.9%. AMA staining was heterogeneous, with high reactivity in oncocytic cells. No significant differences in CaSR, VDR, or AMA expression were found across groups with different hypercalcemia severity. No significant associations were identified between IHC marker expression and parameters of calcium-phosphorus metabolism, as well as cinacalcet treatment response. **Conclusions**: Despite their established roles in parathyroid regulation, CaSR and VDR expression did not correlate with hypercalcemia severity in PHPT. These findings suggest that alternative molecular mechanisms, rather than receptor expression levels, contribute to clinical heterogeneity. Further research is required.

## 1. Introduction

Solitary parathyroid adenomas cause approximately 85–90% of the cases of primary hyperparathyroidism (PHPT) and present well-differentiated, benign, clonal tumors, which cause hypercalcemia through excessive secretion of parathyroid hormone (PTH). Approximately 15–20% of PHPT cases are caused by parathyroid hyperplasia involving multiple glands, whereas multiglandular disease due to double or multiple adenomas is relatively rare; parathyroid carcinoma accounts for approximately 1% of cases [1].

The PHPT severity directly depends on the degree of hypercalcemia, which is the one of the main biochemical markers of the disease. Hypercalcemia due to PHPT can be classified into mild (2.55–2.99 mmol/L), moderate (3–3.49 mmol/L), and severe with high risks of hypercalcemic crisis (more than 3.49 mmol/L). Clinical presentation of increased blood calcium ranges from asymptomatic to renal, skeletal, gastrointestinal and neuromuscular manifestations [2].

The molecular mechanisms explaining the severe course of parathyroid adenomas remain unknown. Most data on larger parathyroid tumors (those with large tumor volume) were associated with severe hypercalcemia and hypercalcemic crisis symptoms, and tumor mass effect. The contribution of distinct parathyroid cell types in tumorigenesis has rarely been considered. A recent study found that oncocytic cell adenomas were associated with higher preoperative serum calcium and PTH levels, as well as a higher incidence of symptomatic disease [3].

The abnormal Ca++ sensing in hyperparathyroidism has been generally attributed to altered calcium-sensing receptor (CaSR) expression. Recent studies employing various methodologies have reported varying levels of both CaSR mRNA and protein expression in parathyroid tumors; reduced CaSR expression likely contributes to impaired PTH release inhibition, but it has been suggested that low levels of G proteins from the Gq subfamily may serve as an additional contributing factor [4].

Parathyroid cells also express the vitamin D receptor (VDR), which additionally affects the expression of the CaSR in parathyroid cells. Reduced VDR mRNA expression has been reported in parathyroid adenomas and may be involved in the pathogenesis of PHPT, rather than resulting from it. The decreased VDR expression can inversely correlate with parathyroid tumor weight, CaSR downregulation and increased secretory set-point. These observations raise the possibility that VDR may be related to the clinically aggressive course of parathyroid adenomas.

Increased secretory activity of parathyroid cells may also be linked to mitochondrial abundance which plays a crucial role in cellular energy metabolism. Mitochondrial dysfunction is frequently associated with various pathological conditions, including tumors. Evidence suggests that the oncocytic parathyroid adenomas are characterized by an elevated mitochondrial count, often accompanied by enlargement and abnormal morphology, which distinguishes them from chief cell adenomas. These alterations may reflect a metabolic shift, potentially contributing to both tumorigenesis and enhanced secretory activity. However, the relationship between the mitochondrial abundance, proliferative activity, and hormonal dysregulation remains to be clarified [5].

The aim of this study was to evaluate the immunohistochemical expression of CaSR, VDR, and antimitochondrial antibodies (AMA) in parathyroid tumors and assess its correlation with the severity of hypercalcemia in patients with primary hyperparathyroidism.

## 2. Materials and Methods

A single-center retrospective study was conducted at the Endocrinology Research Center (ERC), Moscow from January 2018 to August 2023. Clinical trial number: not applicable.

### 2.1. Ethical Considerations and Informed Consent

The study protocol was approved by the Ethics Committee of the Endocrinology Research Center (Protocol No. 1 dated 17 January 2018, Moscow, Russia). The study was conducted in accordance with the principles of the Helsinki Declaration. All participants provided written informed consent for the use of their clinical data and biological samples for research purposes.

The study included 96 patients with PHPT who underwent parathyroidectomy (PTE) at the Center. Based on the severity of hypercalcemia, the patients were divided into four groups: Group 1—Ca corr. ≤ 2.8 mmol/L (*n* = 28); Group 2—2.8 < Ca corr. ≤ 3.0 mmol/L (*n* = 25); Group 3—3.0 ≤ Ca corr. < 3.5 mmol/L (*n* = 29); Group 4—Ca corr. ≥ 3.5 mmol/L (*n* = 14). The use of cut-off points for hypercalcemia was defined according to the Russian PHPT guidelines [6]. Group assignment was based on the baseline albumin-corrected serum calcium documented at diagnosis, prior to the initiation of calcimimetic therapy. Because cinacalcet lowers serum calcium, on-treatment values were not used for stratification; in patients already receiving cinacalcet, the pre-treatment diagnostic calcium was used to define hypercalcemia severity. Furthermore, the degree of hypercalcemia determined the use of cinacalcet either as monotherapy or in combination with other hypercalcemic agents. In combination with cinacalcet, six people received bisphosphonates (3-ibandronic acid, 3-alendronic acid and 1 zoledronic acid), 13 people—denosumab. Importantly, no patient was receiving active treatment with these agents at the time of the baseline calcium measurement used for group stratification. Any such therapy listed here was either initiated subsequently or had been administered in the remote past, well before study enrollment, and thus did not affect the initial assessment of hypercalcemia severity.

Patients underwent several laboratory tests. The ARCHITECT c8000 chemistry analyzer (Abbott, Abbott Park, IL, USA) was used to determine the concentrations of serum calcium (reference interval (RI) 2.15–2.55 mmol/L); albumin (RI 34–48 g/L female, RI 35–50 g/L male); phosphorus (RI 0.74–1.52 mmol/L); creatinine (RI 50–98 µmol/L female, 63–110 µmol/L male); and 24 h urine calcium (RI 2.5–8.0 mmol/L). PTH (range 15–65 pg/mL), osteocalcin (range 11–43 ng/mL female, 14–42 ng/mL male) and β-CrossLaps (range 0.3–1.1 ng/mL female, range 0.1–0.85 ng/mL male) were measured on the COBAS 6000 (Roche, Rotkreuz, Switzerland). Albumin-corrected calcium was calculated using the formula: albumin-corrected calcium (mmol/L) = measured plasma calcium level (mmol/L) + 0.02 × (40 − albumin level (g/L)). eGFR was calculated using the CKD-EPI 2009 formula.

The examination for PHPT complications included dual-energy X-ray absorptiometry (DXA) of the lumbar spine (LS), femoral neck, total femur, and distal third of the radius (Lunar iDXA enCORE, GE Healthcare, Chicago, IL, USA; version 17), with bone mineral density (BMD) assessment performed in accordance with the International Society for Clinical Densitometry (ISCD) guidelines [7], utilizing the T-score for postmenopausal women and men aged 50 and older, and the Z-score for premenopausal women, men under 50; X-ray of the thoracic and lumbar spine (X-ray diagnostic system Optima RF420, GE Healthcare, Chicago, IL, USA) in selected patients based on clinical indications; renal ultrasound (Aplio 500, Toshiba, Tokyo, Japan) and/or renal CT scan (Optima CT660, GE Healthcare, Chicago, IL, USA).

We additionally assessed cinacalcet dosage, tolerability, and its efficacy in reducing hypercalcemia relative to baseline values. The response to cinacalcet was assessed 2–4 weeks after initiation or dose stabilization, using albumin-corrected serum calcium relative to baseline on a stable dose of 30–60 mg/day; a significant response was defined as a reduction of more than 0.25 mmol/L, a partial response as 0.1–0.25 mmol/L, and no effect as less than 0.1 mmol/L.

### 2.2. Morphological Examination

Tumor tissue samples were fixed in 10% buffered formalin, processed in the histological wiring system of Leica ASP6025 (Leica Biosystems, Nussloch, Germany) and poured into paraffin. Further, paraffin sections with a thickness of 3 µm were made from the paraffin-embedded tumor tissue samples on the microtome, applied to slides treated with poly (l-lysine). Then the slides were stained with hematoxylin and eosin according to the standard procedure. All tumor tissue samples were verified in accordance with the 2022 WHO classification of parathyroid tumors [8].

### 2.3. Immunohistochemistry Imaging

**Figure 1:**
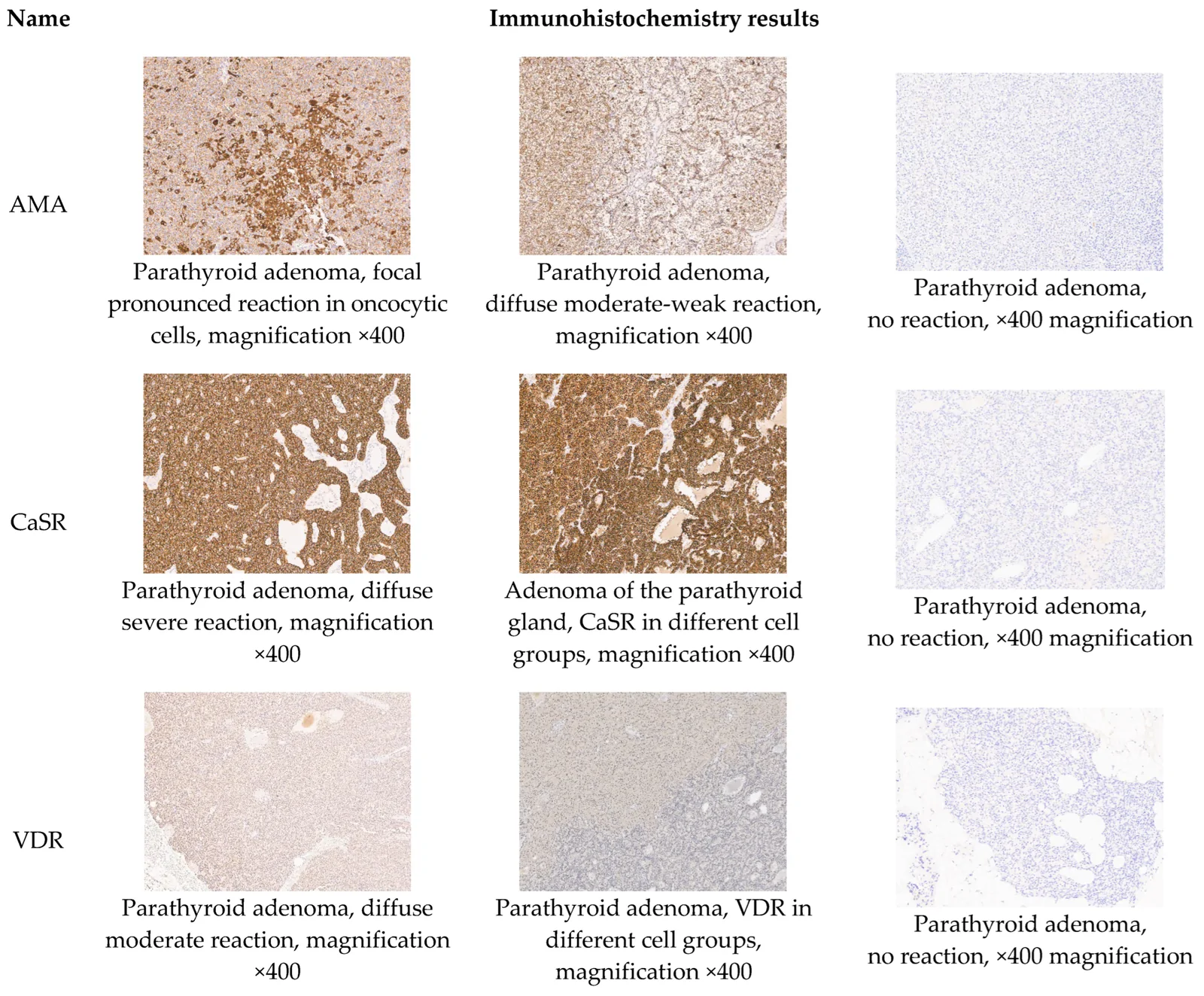
Immunohistochemical staining of parathyroid tumors. Representative immunohistochemical staining patterns of AMA, CaSR, and VDR in parathyroid tumors (×400). Color legend: brown staining indicates positive immunoreactivity for the respective antibodies; blue counterstaining indicates cell nuclei (hematoxylin).

Immunohistochemical analysis of tumor tissue sections was carried out according to the standard technique with a peroxidase detection system with DAB on an automatic Leica BOND III IHC staining system using Leica reagents. Antibodies to vitamin D receptor (VDR, GeneTex, Irvine, CA, USA), calcium-sensing receptor (CaSR, 5C10, GeneTex, Irvine, CA, USA), and anti-mitochondrial antigen (AMA, 113-1, BioGenex, San Jose, CA, USA) were used. All histological slides were scanned using a Leica Aperio AT2 system (Leica Biosystems, Nussloch, Germany) at 20× magnification for further analysis. The most representative fields of view were selected. (Figure 1) CaSR immunostaining was used to assess receptor expression in relation to PTH release; VDR, to test the hypothesized relationship between vitamin-D-receptor expression and CaSR; and AMA, to estimate mitochondrial content in relation to a possible metabolic shift in tumor cells. The following primary antibodies were used: anti-VDR (polyclonal, GeneTex, GTX79289; 1:1000; heat-induced epitope retrieval with BOND ER1, citrate pH 6.0, 20 min), anti-CaSR (mouse monoclonal, clone 5C10, IgG2a, GeneTex, GTX19347; 1:150; BOND ER2, EDTA pH 9.0, 10 min) and anti-AMA (mouse monoclonal, clone 113-1, IgG1, BioGenex, MU213-UC; 1:200; BOND ER2, EDTA pH 9.0, 10 min), with the BOND Polymer Refine Detection System (DAB). Kidney tissue served as a positive control for CaSR and VDR and liver tissue for AMA. As a negative control, the primary antibody was replaced with antibody diluent; areas of dense fibrous connective tissue served as an internal negative tissue control. Isotype-matched controls were not available and are planned for future work. The specificity of clone 5C10 in parathyroid and renal tissue is supported by prior reports [9,10,11]; reporting follows recognized IHC validation standards [12] and REMARK recommendations.

The expression of AMA and VDR was determined in the cytoplasm of tumor cells and evaluated as strong (1), moderate (2), weak or absent (3). CaSR expression was detected in the membrane of tumor cells and evaluated similarly. Immunostaining was evaluated by a single experienced endocrine pathologist, blinded to the hypercalcemia group and clinical data. The three-tier intensity scale was adapted from commonly used semiquantitative IHC grading rather than developed de novo. To provide a quantitative measure, an H-score (0–300) was additionally calculated for each case as H-score = 0 × f0 + 1 × f1 + 2 × f2 + 3 × f3, where f0–f3 are the proportions of cells at each intensity; at least 10 high-power fields (×400; field diameter 0.54 mm) were assessed per sample. As scoring was performed by a single observer, interobserver agreement could not be assessed. Assessment of normal parathyroid tissue was not an objective of the study; where unchanged parathyroid tissue was incidentally present in the same section it served only as an internal semiquantitative reference for relative staining intensity, and no separate H-score was calculated for it, so no systematic per-patient tumor-versus-normal comparison was performed.

### 2.4. Statistical Analysis

Statistical analysis was performed using the Statistica v. 13.3 software package (TIBCO Software Inc., Palo Alto, CA, USA). For the description of quantitative data, medians and interquartile ranges were used, presented as Me [Q1; Q3], while absolute and relative frequencies were used for qualitative data. The comparison of four independent groups for quantitative indicators was carried out using the Kruskal–Wallis test, followed by post hoc analysis. Intergroup differences in categorical variables were assessed using the Freeman–Halton test. The significance level (*p*) for testing statistical hypotheses was set at 0.05. To correct the critical significance level for multiple comparisons, the Bonferroni correction (p0) was applied, after which *p*-values in the range between the calculated p0 and 0.05 were interpreted as a statistical trend.

## 3. Results

The study included 86 women and 10 men, with a median age of 57 years [47.5; 67] at the time of enrollment. The majority of patients presented with symptomatic PHPT with either bone or renal complications. A detailed description is provided in Table 1. End-organ involvement is detailed in Table 2. Cinacalcet was administered in a median dose of 30 mg/day [30; 60] prior to surgery in 73.9% of cases. Exceptions included 22/28 patients in the mild hypercalcemia subgroup and 2/14 patients in the life-threatening hypercalcemia group. Treatment tolerance was satisfactory in 91.7% of cases. A significant response to cinacalcet therapy at average doses of 30–60 mg per day was noted in 35.7% of patients (defined as a reduction in calcium levels of more than 0.25 mmol/L from baseline), while no effect was observed in 42.9%.

The histological examination revealed predominated adenomas, while atypical tumors and carcinoma of the parathyroid gland were found in 10.4% and 2.1% of cases, respectively. The diagnosis of hyperplasia was established only in one patient (Table 3). In the group with Ca corr. ≥ 3.5 mmol/L an atypical tumor was noted in one person, in the group 3.0 ≤ Ca corr. < 3.5—in four patients. Among patients with 2.8 < Ca corr. ≤ 3.0, there were two atypical tumors and two carcinomas, and three atypical tumors were established in the group with Ca corr. < 2.8 mmol/L.

In our study, the vast majority of adenomas exhibited a mixed cellular composition. Chief cell-only adenomas were observed in 18.75% (18/96) of cases, while clear cell-only adenomas were rare, accounting for only 1% (1/96). No adenomas composed exclusively of oncocytic cells were identified. Among Group 1, chief cells predominated in 53.6% (15/28) of adenomas, while clear cells were the dominant type in 25% (7/28). In Group 2, chief cell adenomas were identified in 24% (6/25) of tumors, clear cell adenomas in 24% (6/25) and oncocytic cell adenomas in 8% (2/25). In Group 3, chief cells predominated in 48.3% (14/29) of adenomas, clear cells in 10.3% (3/29), and oncocytic adenomas accounted for 3.4% (1/29). Finally, in Group 4, chief cell adenomas accounted for 28.6% (4/14) of cases, pure cell adenomas predominated in 14.3% (2/14), and predominantly oncocytic adenomas were identified in 7.1% (1/14) of samples. We did not find any associations between serum calcium levels and predominant adenoma histological type (all *p* > 0.05; *p* = 0.137, *p* = 0.238, and *p* = 0.276 for chief cells, water-clear cells, and oncocytic cells, respectively) (Table 4).

Almost all parathyroid lesions demonstrated pronounced strong expression of CaSR, comparable to that of adjacent unchanged parathyroid tissue, where present. All tumors (96/96, 100%) showed positive immunoreactivity for CaSR, with strong staining in 92/96 (95.8%) and moderate staining in 4/96 (4.2%); no tumor was negative. Membrane-cytoplasmic staining was detected in 86.5% (83/96) of parathyroid tumor samples, whereas pure cytoplasmic staining was observed in 11.5% (11/96) of cases. Decreased VDR expression was observed in 46.9% of cases, where tumor cells were less intensely stained than the adjacent unchanged parathyroid tissue, where present. Anti-mitochondrial antibodies showed heterogeneous “mosaic” expression, with alternating cells exhibiting strong and weak cytoplasmic staining throughout the examined material. When compared with histological data, it was found that the cytoplasm of oxyphilic cells was stained more intensely than that of chief and clear cells. In a separate subgroup of atypical tumors, four out of 10 samples showed focal or weak expression of VDR, and the expression of AMA also decreased in five out of 10 samples. The study group included only two carcinomas, in both cases, the expression of VDR was focal, which distinguished it from healthy tissue.

Further, we conducted a comparative analysis of groups 1–4. As expected, the groups differed in levels of iPTH and bone remodeling markers. Among the clinical features statistically significant differences were found only for the frequency of fibrocystic osteitis, which was higher in patients with Ca corr. more than 3.5 mmol/L. A significant decrease in BMD and impaired renal filtration function showed a statistical trend that was more pronounced in patients with hypercalcemia greater than 3 mmol/L (*p* = 0.020 and *p* = 0.002, respectively, p0 (Bonferroni correction) = 0.0017).

We performed an analysis of associations to assess the relationship between the degree of IHC and key indicators of calcium-phosphorus metabolism; however, no significant correlations were identified. Additionally, no association was found between the expression of CaSR and VDR and the effectiveness of the cinacalcet therapy.

Quantitative H-score analysis was available for 95 of 96 patients. Median [Q1; Q3] H-scores by hypercalcemia-severity group (Ca corr. ≤ 2.8; 2.8–3.0; 3.0–3.5; ≥3.5 mmol/L) were: VDR 73 [53; 110], 75 [45; 109], 107 [59; 119], 67 [40; 100] (*p* = 0.38); CaSR 300 [300; 300] in all groups (*p* = 0.66); AMA 109 [101; 119], 108 [81; 127], 106 [65; 125], 207 [58; 239] (*p* = 0.39; Kruskal–Wallis). CaSR H-score was maximal (300) in 92 of 95 tumors, confirming a pronounced ceiling effect. In a sensitivity analysis restricted to histologically confirmed adenomas (excluding atypical tumors [*n* = 10], hyperplasia [*n* = 1] and carcinomas [*n* = 2]), no significant differences in VDR, CaSR or AMA H-score across severity groups were observed. Overall, quantitative expression of CaSR, VDR and AMA was not associated with hypercalcemia severity. The distribution of H-scores by severity group for all three markers is shown in Supplementary Figure S1, which illustrates both the ceiling effect for CaSR and the overlapping distributions for VDR and AMA.

## 4. Discussion

Parathyroid cells in vivo and in vitro respond to increasing concentrations of extracellular ionized calcium by reducing PTH secretion. This effect is mediated by a direct interaction of calcium with a G protein-coupled CaSR. In patients with PHPT high concentrations of ionized calcium do not suppress autonomous PTH secretion, but the molecular mechanism for this abnormality is not clearly understood [13]. CaSR down-regulation is generally believed to be the principal reason for the abnormal calcium-PTH interactions in parathyroid tumors, i.e., calcium responsiveness should be directly dependent upon CaSR expression on cells. Corbetta et al. reported diminished CaSR expression both at mRNA (by RT-PCR) and protein level (by Western blot) in 27 parathyroid adenoma and four hyperplasia cases. The authors also proposed that the disrupted response of parathyroid cells to extracellular calcium concentrations in adenomas might be associated with a 2-fold reduction in the expression of the membrane G-protein [4]. Singh et al. [14] using real-time qPCR revealed reduced CaSR mRNA expression with a fold reduction of 0.12 (*p* < 0.0001) in parathyroid adenomas. The obtained data were correlated with the results of IHC results showing reduced expression of CaSR in 90% of adenomas. Unlike the findings of Singh et al., we did not observe a significant reduction in CaSR expression in different tumors compared to healthy tissue. The reaction with CaSR antibodies predominantly exhibited diffuse and pronounced membrane-cytoplasmic expression, which was almost similar to intact parathyroid tissue. Moreover, strong positive IHC reactions persisted even in cases of extremely “aggressive” PHPT (including carcinomas and atypical tumors) and hypercalcemia exceeding 3.5 mmol/L. At the same time, Haven et al. did not find a significant decrease in CaSR expression in parathyroid hyperplasia or adenomas. While only one of 104 benign tumors (1%) exhibited irregular or absent CaSR staining, carcinomas showed loss of CaSR expression in 31% of cases (9/29) [15].

In Agarwal et al.’s study [16], normal autopsy-derived parathyroid tissues showed strong but predominantly incomplete membranous CaSR expression (in 85% of cases). The cytoplasm of the normal parathyroid cells also showed strong expression of CaSR. In the PHPT group, membranous staining for CaSR was usually of moderate intensity. In addition, none of the parathyroid hyperplasia cases showed moderate cytoplasmic staining as the predominant pattern, in contrast to 16% of adenomas and 43% of the carcinomas. The authors suggested that not only diminished CaSR synthesis, but additional defects in the tracking of the receptor from the cytoplasm to the membrane may play a role in the PHPT pathogenesis. In our series, pure cytoplasmic CaSR staining was identified in 11.5% of tumors. While membranous expression is considered canonical, cytoplasmic localization has been documented in large-scale subcellular mapping efforts [17,18]. Beyond surface signaling, CaSR undergoes dynamic trafficking (ER–Golgi–plasma membrane), endocytosis, and proteasomal/lysosomal processing, processes that can yield intracellular receptor pools detectable by IHC [19]. Moreover, recent work highlights CaSR-mediated intracellular communication among the endoplasmic reticulum, mitochondria, and nucleus [20]. Experimental studies also indicate ER-linked regulation of CaSR processing and surface expression [21]. Taken together, these data suggest that our cytoplasmic CaSR staining may reflect receptor trafficking and intracellular signaling rather than solely nonspecific staining; however, standardized protocols and orthogonal validation will be required to define its biological significance in parathyroid tumors [22].

Interesting data was obtained by Shi Y. et al. Using flow cytometric analysis of resected adenomatous parathyroid glands, they have isolated and characterized chief and oncocytic cells, as well as tumor-infiltrating lymphocytes. While chief and oncocytic cells exhibited comparable CaSR expression at both protein and mRNA levels, oncocytic cells demonstrated greater calcium sensitivity despite showing higher basal PTH secretion. This suggests that CaSR expression alone does not determine PTH production and calcium responsiveness in the parathyroid gland. Increased PTH levels in PHPT may reflect not only the total number of tumor cells but also differences in secretory profiles among distinct cell types. Previous studies have suggested that oxyphil cells can demonstrate a higher secretory activity compared to chief cells. However, in our cohort, purely oncocytic adenomas were not observed, and oxyphil-predominant tumors were rare. Most adenomas displayed either mixed cellular composition or predominance of chief cells. Therefore, while oxyphil cells may potentially contribute to more intensive PTH secretion, their overall impact on circulating PTH levels in PHPT is likely limited by their relatively low representation in most tumors. The authors further proposed that tumor-infiltrating lymphocytes might target antibodies against CaSR, PTH, or other parathyroid antigens. Such antibody-mediated mechanisms—through either CaSR inhibition or immune clearance of CaSR-expressing/PTH-producing cells—could initially trigger compensatory hyperplasia, potentially progressing to adenoma formation [23]. In our work, the majority of adenomas exhibited a mixed cellular composition. Furthermore, there were no associations between serum calcium levels and adenoma histological type.

With regard to VDR, we obtained more expected results. The VDR expression was reduced or absent in approximately 50% of cases, which is generally consistent with previously published research. Carling et al. demonstrated similar VDR mRNA levels in the adenomas and hyperplasias, which were reduced (42 ± 2.8% and 44 ± 4.0%) compared to the normal glands (*p* < 0.0001) [24]. Rao et al. evaluated receptor expression by IHC in 24 parathyroid adenomas and in 11 normal glands. There was reduction in VDR expression in almost all cells of parathyroid adenomas. The decrease in CaSR expression was not so pronounced as VDR, which generally does not contradict our data. The authors proposed that reduced CaSR expression may frequently occur secondary to VDR loss. While this downregulation may be moderate, it appears sufficient to elevate the PTH secretory set-point and subsequently promote adenoma development [25]. This hypothesis is supported by the presence of vitamin D response elements (VDREs) in the 5′-flanking region of the *CASR* gene, enabling its transcriptional regulation through 1,25-dihydroxyvitamin D3-VDR complex binding to these regulatory sequences [26]. Because normal parathyroid tissue was assessed only opportunistically, as an internal reference, rather than systematically, our data do not permit a formal quantitative tumor-versus-normal comparison; this methodological difference should be considered when comparing our findings with studies specifically designed to quantify expression in normal glands and may partly explain the apparent discrepancies with previous reports.

Data on the AMA expression in the parathyroid tumors are limited. We observed heterogeneous expression of the AMA throughout the studied samples. It was noted that the cytoplasm of oncocytic cells exhibited more intense staining compared to chief and clear cells. The most likely reason for this reaction is the presence of a high number of mitochondria in the cytoplasm of oncocytic cells. In contrast, cells with clear cytoplasm displayed the weakest staining, with some cases showing no staining at all. The origin of these clear cells remains insufficiently studied. There is a hypothesis that clear cytoplasm cells represent the final stage of chief cell hyperplasia. Ultrastructural analysis of this cell type has shown that vacuoles may either be dilated structures of the Golgi complex or components of the endoplasmic reticulum, which could explain the observed IHC reactions with AMA. For a more accurate assessment, further analysis on a larger sample size is necessary [27]. The anti-mitochondrial antibody (clone 113-1) recognizes a 60 kDa non-glycosylated protein component of human mitochondria and produces a granular cytoplasmic pattern; it was used as a structural marker of mitochondrial content. AMA staining should therefore be interpreted as a surrogate marker of mitochondrial abundance (mass) rather than a direct measure of mitochondrial function or metabolic activity, and it was not validated against electron microscopy or mtDNA quantification; this proxy nature is acknowledged as a limitation.

In the study by Varshney S. et al., the expression of CaSR and VDR in parathyroid adenomas was lower than in normal tissue (according to IHC analysis and mRNA expression). No correlations were identified between CaSR and VDR expression and preoperative levels of calcium, iPTH, and 25(OH)D. Other markers including PTH and CD1 also did not correlate with serum calcium and iPTH. Similar results were obtained in our study. We found no differences in the expression of CaSR, VDR, and AMA among groups with varying severity of hypercalcemia, as well as no associations between the IHC phenotype and the response to calcimimetic therapy. The assessment of the immunohistochemical profile of parathyroid tumors in relation to the severity of hypercalcemia has been conducted for the first time [28].

### Strengths and Limitations

This study is, to our knowledge, the first to investigate the relationship between CaSR, VDR, and AMA expression and the severity of hypercalcemia in PHPT. The relatively large cohort with detailed clinical and biochemical data is a strength. Limitations include the retrospective, single-center design and the small number of non-adenomatous tumors, which prevented separate analysis of these subgroups. Future prospective studies are needed to explore additional molecular mechanisms underlying clinical variability in PHPT.

Additional limitations include the inclusion of different histological types of parathyroid tumors (adenomas, atypical tumors, carcinomas), which, given their distinct biology, may have influenced the results. The lack of data on resected parathyroid gland volume and baseline patient 25(OH)D levels also precludes assessment of their potential impact on hypercalcemia severity and the expression of the studied markers. In addition, CaSR expression showed minimal variability across the cohort (strong in 92/96 tumors, with no weak or absent cases), which limits the statistical power of the across-group comparison; the absence of a difference in CaSR expression between severity groups should therefore be interpreted with caution. Resected gland weight and volume were not systematically recorded and could not be adjusted for, so residual confounding by tumor burden cannot be excluded. Baseline 25(OH)D was available for 36 of 96 patients (median 19.0 ng/mL, IQR 12.6–30.5), indicating frequent vitamin D deficiency. Within this subgroup, 25(OH)D did not differ between tumors with preserved versus reduced VDR expression (median 20.1 vs. 17.5 ng/mL; *p* = 0.89) and showed no correlation with the VDR H-score (Spearman rho = 0.01, *p* = 0.97). Although vitamin D status was not available for the whole cohort, these data suggest it is unlikely to fully explain the VDR findings; prospective assessment in all patients remains warranted, and these results should be interpreted with caution given the limited sample. Owing to the pronounced ceiling effect, CaSR could not be meaningfully compared across severity groups, and the absence of association should not be interpreted as evidence that CaSR is unrelated to severity. Hypercalcemia severity in PHPT is multifactorial and may be influenced by PTH concentration, renal function, histological subtype, prior treatment, tumor size or weight, and vitamin D status. Given the cohort size (*n* = 96) unevenly distributed across four severity groups, and because several of these variables were unrecorded (gland weight or volume) or available only in a subset (25(OH)D, *n* = 36), a multivariable analysis was not statistically justifiable and would be prone to overfitting; it was therefore not performed. This is a limitation of the present exploratory study, and these confounders should be incorporated in larger prospective cohorts. Quantitatively, applying the conventional rule of thumb of at least 10 observations per candidate variable, the limiting stratum (Group 4, *n* = 14) supports approximately one covariate (14/10 = 1.4), whereas the confounders identified above comprise at least six to seven variables, which would require roughly 70 patients per severity group (approximately 280 in total). Moreover, a complete-case model including 25(OH)D would reduce the analyzable sample to 36 patients (about nine per group), yielding fewer than one observation per variable. The available data therefore fall short of the minimum required for stable multivariable estimation by an order of magnitude.

The study was not designed to evaluate predictors of low BMD such as age, severity of hypercalcemia, or other clinical variables, and therefore subgroup analyses addressing these associations were not performed.

## 5. Conclusions

CaSR expression remained strong in nearly all tumors, including aggressive cases. Because this uniformly high expression left virtually no variance (a ceiling effect), our data could not evaluate an association between CaSR expression and hypercalcemia severity; this question remains open and should be addressed with quantitative methods in cohorts with greater expression variability. In contrast, VDR expression was diminished in approximately half of the cases, aligning with previous research implicating VDR downregulation in PHPT pathogenesis. AMA expression exhibited heterogeneity, with oncocytic cells showing more intense staining, likely due to their mitochondrial-rich cytoplasm. No significant correlations were found between the IHC profiles of CaSR, VDR, or AMA and the degree of hypercalcemia, bone remodeling markers, or response to cinacalcet therapy. These results suggest that other molecular mechanisms, beyond the examined markers, may contribute to the clinical variability of PHPT. Future studies incorporating parathyroid functional imaging (e.g., 99mTc-sestamibi or 18F-choline PET/CT) could provide additional insights by correlating metabolic tumor activity with the immunohistochemical profile and clinical phenotype. Further research is warranted to explore additional pathways and cellular interactions underlying the disease’s heterogeneity.

## Figures and Tables

**Table 1:** Clinical and biochemical characteristics of the study cohort. PTH, parathyroid hormone; eGFR, estimated glomerular filtration rate. DXA was available in 86 of 96 patients; the 28 patients with a reported median Z-score are a subset of these 86.

Sign		*N*	Me [Q_1_; Q_3_]/*n* (%)
Sex	Female	96	86 (89.6%)
	Male		10 (10.4%)
Age at diagnosis, years		96	57 [47.5; 67]
PTH, pg/mL		96	248.55 [138.15; 558.50]
Phosphorus, mmol/L		92	0.78 [0.665; 0.93]
Daily calciuria, mmol/D		88	8.70 [6.095; 10.965]
eGFR (CKD-EPI), mL/min/1.73 m^2^		96	81.5 [64.0; 97.5]

**Table 2:** End-organ involvement in the study cohort. DXA was available in 86 of 96 patients.

End-Organ Involvement	Category	*N*	*n* (%)/Me [Q1; Q3]
Bone mineral density (DXA Z-scores), SD		28	−1.45 [−2.35; −0.75]
Bone status by DXA (T-scores)	Normal (T-score ≥ −1.0/Z-score ≥ −2.0)	86	24 (27.9%)
	Osteopenia (−1.0 > T-score > −2.5)	86	18 (20.9%)
	Osteoporosis (T-score ≤ −2.5/Z-score < −2.0)	86	29 (33.7%)
	Severe osteoporosis (T-score ≤ −2.5/Z-score < −2.0 + low-energy fracture)	86	15 (17.4%)
Vertebral fractures		96	19 (19.8%)
Non-vertebral low-energy fractures		96	14 (14.6%)
Nephrocalcinosis/nephrolithiasis		96	68 (70.8%)

**Table 3:** Histological features of parathyroid tumors.

Characteristic		*N*	Me [Q_1_; Q_3_]/*n* (%)
**Histology**	Adenoma	96	83 (86.5%)
	Atypical tumor		10 (10.4%)
	Hyperplasia		1 (1%)
	Carcinoma		2 (2.1%)
**CaSR expression**	1	96	92 (95.8%)
	2		4 (4.2%)
	3		0 (0%)
**VDR expression**	1	96	51 (53.1%)
	2		35 (36.5%)
	3		10 (10.4%)
**AMA expression**	1	96	46 (47.9%)
	2		47 (49%)
	3		3 (3.1%)

1—strong expression, 2—moderate expression, 3—weak or absence expression.

**Table 4:** Predominant adenoma cell type by hypercalcemia-severity group (Ca corr., mmol/L); no significant association was found between serum calcium and predominant cell type (all *p* > 0.05). Patients were divided into four groups according to albumin-corrected serum calcium levels at study inclusion: Group 1 (Ca corr ≤ 2.8 mmol/L), Group 2 (2.8 < Ca corr ≤ 3.0 mmol/L), Group 3 (3.0 ≤ Ca corr < 3.5 mmol/L), and Group 4 (Ca corr ≥ 3.5 mmol/L). The figure illustrates the proportion of adenomas composed predominantly of chief cells, clear cells, or oncocytic cells in each group.

Predominant Cell Type	Group 1 (≤2.8; *n* = 28)	Group 2 (2.8 < Ca corr. ≤ 3.0; *n* = 25)	Group 3 (3.0 ≤ Ca corr. < 3.5; *n* = 29)	Group 4 (≥3.5; *n* = 14)	*p*
Chief cells	53.6% (15/28)	24.0% (6/25)	48.3% (14/29)	28.6% (4/14)	0.137
Clear (water-clear) cells	25.0% (7/28)	24.0% (6/25)	10.3% (3/29)	14.3% (2/14)	0.238
Oncocytic cells	0% (0/28)	8.0% (2/25)	3.4% (1/29)	7.1% (1/14)	0.276

## Data Availability

The datasets used and/or analyzed during the current study are available from the corresponding author on reasonable request.

## Supplementary Materials

The following supporting information can be downloaded at: https://www.mdpi.com/article/10.3390/cimb48080766/s1.
