# Supplementary material for: Immunohistochemical Expression of CaSR, VDR, and AMA in Parathyroid Tumors in Relation to Hypercalcemia Severity in Primary Hyperparathyroidism
Source: Curr Issues Mol Biol. 2026 Jul 28;48(8):766. doi: 10.3390/cimb48080766 (PMC13510422; doi:10.3390/cimb48080766)
Supplement: Supplementary file 1 [file cimb-48-00766-s001.zip › cimb-4391352-supplementary.pdf]

Supplementary Materials

Immunohistochemical Expression of CaSR, VDR, and AMA in Parathyroid Tumors and Hypercalcemia Severity in Primary Hyperparathyroidism

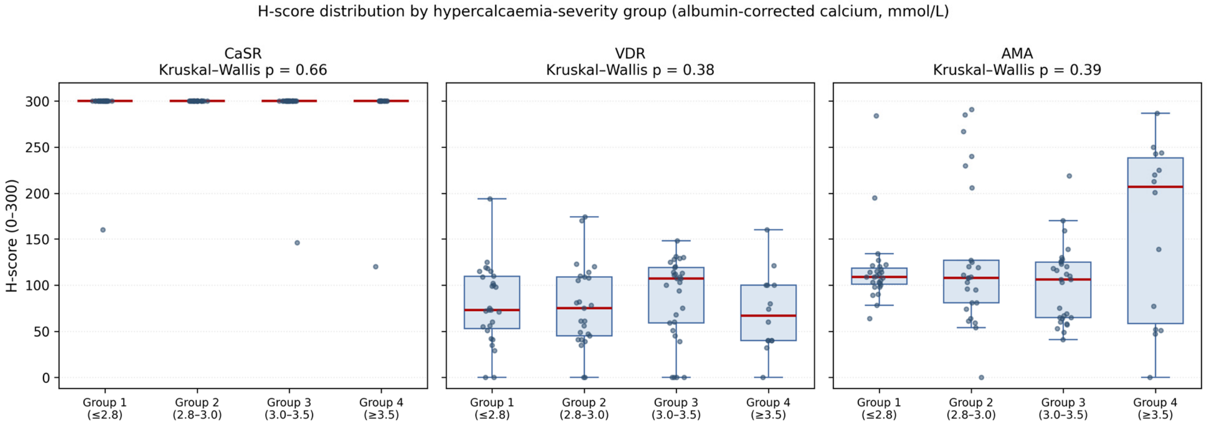

Figure S1: H-score distribution by hypercalcaemia-severity group (albumin-corrected serum calcium, mmol/L) for CaSR, VDR, and AMA. Groups: Group 1, Ca corr. <= 2.8 mmol/L (n = 27); Group 2, 2.8 < Ca corr. <= 3.0 mmol/L (n = 25); Group 3, 3.0 <= Ca corr. < 3.5 mmol/L (n = 29); Group 4, Ca corr. >= 3.5 mmol/L (n = 14). Boxes show the median and interquartile range, whiskers the 1.5x interquartile range, and dots individual patients (n = 95 with available H-score). CaSR shows a pronounced ceiling effect, with a median H-score of 300 and an interquartile range of 0 in every group. p values are from the Kruskal-Wallis test across the four severity groups; no marker differed significantly between groups.
